# Playful Minds: Using Improvisational Theatre to Enhance Cognitive Functioning in Older Adulthood

**DOI:** 10.1093/geroni/igaf122.1807

**Published:** 2025-12-31

**Authors:** Shoshi Keisari, Yulia Golland, Boaz Ben-David

**Affiliations:** University of Haifa, Haifa, HaZafon, Israel; Reichman University, Herzliya, HaMerkaz, Israel; Reichman University, Herzliya, Tel Aviv, Israel

## Abstract

Social playfulness, characterized by spontaneity and mutual enjoyment, allows individuals to step away from routine roles and engage in novel and surprising exchanges. Emerging evidence suggests that social playfulness is a promising approach for supporting cognitive functions in aging in a joyful and engaging way. This study examined how brief playful interactions that are based on improvisational theatre-based interactions enhance cognitive functions in older adults. Across three experimental studies, 150 participants aged 75 to 100 engaged in two types of interactions: playful interactions and a control activity (e.g., structured conversations or routine exercises). Sessions were conducted either face-to-face or online (Zoom). Cognitive tests and self-report measures were administered before and after each interaction. Findings indicate that brief playful interactions improved cognitive functions, including digit span performance, word fluency, and response times on the Flanker test, compared to control activities. Playful interactions also enhanced social closeness and increased positive emotions. Rooted in drama therapy principles, improvisational theatre offers a natural, engaging alternative to traditional cognitive training. Unlike structured cognitive interventions, playful interactions tap into spontaneity, co-creation, and embodied engagement, making them both enjoyable and accessible. These findings suggest that integrating playful interactions into routine care could offer a simple, scalable approach to supporting cognitive health in older adults.

